# The Telomere Capping Complex CST Has an Unusual Stoichiometry, Makes Multipartite Interaction with G-Tails, and Unfolds Higher-Order G-Tail Structures

**DOI:** 10.1371/journal.pgen.1003145

**Published:** 2013-01-03

**Authors:** Neal F. Lue, Ruobo Zhou, Lidia Chico, Ninghui Mao, Olga Steinberg-Neifach, Taekjip Ha

**Affiliations:** 1 Department of Microbiology and Immunology, W. R. Hearst Microbiology Research Center, Weill Medical College of Cornell University, New York, New York, United States of America; 2 Department of Physics and Center for the Physics of Living Cells, University of Illinois at Urbana-Champaign, Urbana, Illinois, United States of America; 3 Howard Hughes Medical Institute, Urbana, Illinois, United States of America; 4 Hostos Community College, City University of New York, Bronx, New York, United States of America; University of California Berkeley, United States of America

## Abstract

The telomere-ending binding protein complex CST (Cdc13-Stn1-Ten1) mediates critical functions in both telomere protection and replication. We devised a co-expression and affinity purification strategy for isolating large quantities of the complete *Candida glabrata* CST complex. The complex was found to exhibit a 2∶4∶2 or 2∶6∶2 stoichiometry as judged by the ratio of the subunits and the native size of the complex. Stn1, but not Ten1 alone, can directly and stably interact with Cdc13. In gel mobility shift assays, both Cdc13 and CST manifested high-affinity and sequence-specific binding to the cognate telomeric repeats. Single molecule FRET-based analysis indicates that Cdc13 and CST can bind and unfold higher order G-tail structures. The protein and the complex can also interact with non-telomeric DNA in the absence of high-affinity target sites. Comparison of the DNA–protein complexes formed by Cdc13 and CST suggests that the latter can occupy a longer DNA target site and that Stn1 and Ten1 may contact DNA directly in the full CST–DNA assembly. Both Stn1 and Ten1 can be cross-linked to photo-reactive telomeric DNA. Mutating residues on the putative DNA–binding surface of *Candida albicans* Stn1 OB fold domain caused a reduction in its crosslinking efficiency *in vitro* and engendered long and heterogeneous telomeres *in vivo*, indicating that the DNA–binding activity of Stn1 is required for telomere protection. Our data provide insights on the assembly and mechanisms of CST, and our robust reconstitution system will facilitate future biochemical analysis of this important complex.

## Author Summary

Telomeres are the special structures that protect the chromosomal ends against aberrant rearrangements. The most distal portion of a telomere is bound by the CST (Cdc13-Stn1-Ten1) complex, which is critical for telomere protection. By preparing and analyzing large quantities of the CST complex derived from a fungus called *Candida glabrata*, we showed that CST has an intricate arrangement and each complex probably contains 2, 4 (or 6), and 2 copies of the Cdc13, Stn1, and Ten1 protein, respectively. By investigating the interaction between pairs of subunits, we provide support for the notion that Stn1 acts as a bridge to bring the three components together. In addition, using a technique that measures the properties of individual fluorescent DNA molecules, we demonstrated that telomere single-stranded DNA has a tendency to fold back on itself, but that the binding of the CST complex to DNA disrupts the folding and makes the DNA more extended. We also showed that Stn1 can touch DNA directly and that this property is required for Stn1 to protect telomeres in the cell. Finally, we found that CST can bind DNA that carries non-telomeric sequences, suggesting the complex may play a role at other chromosomal locations.

## Introduction

The special nucleoprotein structures located at the ends of linear eukaryotic chromosomes, known as telomeres, are critical for chromosome stability; they protect the terminal DNAs from degradation, end-to-end fusion, and other abnormal transactions [1]–[3]. In most organisms, telomeric DNA consists of short G-rich repeats that terminate in 3′ overhangs referred to as G-tails. The G-tails of budding yeast are bound by CST (Cdc13-Stn1-Ten1), a complex that is critical for both telomere protection and replication [4]–[7]. Genetic and structural analyses suggest that CST exhibits many feature of the RPA complex, which constitutes the major single-strand DNA-binding complex in eukaryotes [5], [6], [8]. Interestingly, even though CST proteins were initially discerned only in budding yeast, recent studies have uncovered Stn1 and Ten1 homologues in fission yeast, as well as CST-like complexes in plants and mammals, indicating that this complex is widely conserved [9]–[11]. Notably, a Stn1 homologue named *Verrocchio* is now known to be critical for telomere stability in *Drosophila*, an organism that utilizes retrotransposons rather than short repeats to cap chromosome ends [12], [13]. CST components thus appear to be universally present and involved in telomere regulation. In support of this idea, the human CST complex was recently shown to regulate telomerase activity, telomere replication, and telomere C-strand synthesis [14]–[17].

Among all the CST complexes, the structures and mechanisms of the *S. cerevisiae* subunits are the most extensively characterized. *Sc*Cdc13 is a large multifunctional protein that contains probably four OB fold domains and that binds to nucleic acid as well as protein targets. The first, third and last OB fold domains of Cdc13 (named OB1, DBD and OB4) are known to interact with Pol1 (the catalytic subunit of Pol α), G-tail, and Stn1, respectively [18], [19]. The OB1 domain also mediates dimerization, which in turn creates an extended surface for Pol1 binding [18]. Moreover, the recruitment domain (RD) of Cdc13, located between OB1 and OB2, binds to the telomerase subunit Est1 to promote the recruitment of telomerase to chromosome ends and the activation of telomerase [20]–[22]. In comparison to *Sc*Cdc13, fewer interaction partners have been identified for *Sc*Stn1 and *Sc*Ten1. *Sc*Stn1 and *Sc*Ten1 together form a stable subcomplex and each protein has been reported to bind telomere G-strand with moderate to low affinity [5], [23]. *Sc*Stn1 is also known to interact with Pol12, a regulatory subunit of the Pol α complex [24]. The multiplicity of interactions between CST and Pol α supports a role for CST in regulating telomere C-strand synthesis, which is presumed to be mediated by pol α [7].

Analyses of CST homologues or analogues in diverse organisms have revealed intriguing structural and mechanistic variations in these critical telomere capping proteins. For instance, CTC1s (the largest subunit of the mammalian and plant complex), despite having multiple OB fold domains, exhibit little sequence similarity to Cdc13 and possess no autonomous DNA-binding activity [10]. Unlike *Sc*Cdc13, the mammalian CST complex manifests distinct recognition properties for short and long single stranded (ss) DNA; it binds preferentially to short oligonucleotides (<20 nts) with G-strand repeats, but indiscriminately to long oligonucleotides (>50 nts) with a wide variety of sequences [10], [14], [25]. Other instances of striking variation are found in many *Candida* species, where the Cdc13 orthologues are quite small and consist of just the DBD and OB4 domains [6], [26]. Notwithstanding the absence of the OB1 domain, these orthologues nevertheless form dimers through an alternative interface involving specialized loops in their OB4 regions [26]. Moreover, in contrast to *Sc*Cdc13, dimerization of *Candida* Cdc13s is required for high affinity and sequence-specific recognition of telomeric DNA [26]. These observations raise fascinating questions concerning the mechanistic diversity and evolutionary plasticity of the CST complex.

Not withstanding considerable knowledge on the structure and function of fungal CST subunits, studies of the complex has been hampered by an inability to reconstitute and isolate adequate quantities of the full complex for detailed biochemical investigations. Thus, the precise assembly mechanisms of the complex (e.g., how the subunits interact with one another) remain undefined. Whether the incorporation of the Stn1 and Ten1 subunit alters the DNA-binding property of Cdc13 is likewise unclear. To address such deficiencies, we systematically screened CST homologues for co-expression and complex assembly in *E. coli*. After considerable trials, we developed a strategy for isolating large quantities of the *Candida glabrata* CST complex, which was shown to have an unusual stoichiometry. Both Cdc13 and the CST complex were found to recognize G-tails with high affinity and sequence-specificity, and to be capable of unfolding higher order G-tail structures. Additional studies suggest that Stn1 and Ten1 can contact DNA directly in the context of the full CST-DNA assembly. Mutating residues on a hypothesized DNA-binding surface of *Candida albicans* Stn1 OB fold domain caused a reduction in its DNA-binding *in vitro* (as measured by a photo-crosslinking assay) and engendered long and heterogeneous telomeres *in vivo*, supporting the physiologic significance of DNA-binding by Stn1.

## Results

### The *C. glabrata* CST complex has an unusual stoichiometry

**Figure 1:**
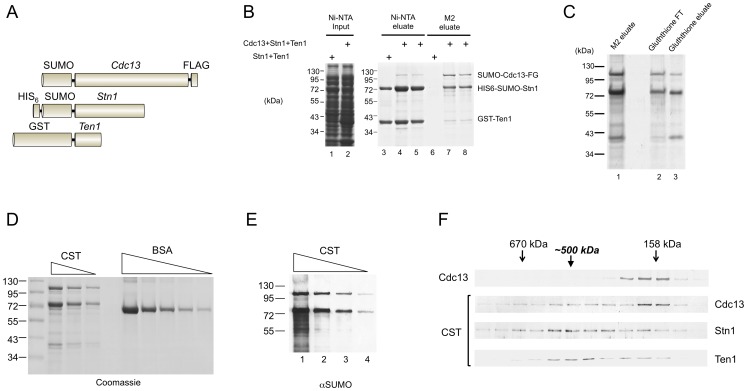
Purification and characterization of the *C. glabrata* CST complex. (A) The fusion proteins co-expressed in *E. coli* are schematically illustrated. (B) Extracts were isolated from *E. coli* strains co-expressing Stn1 and Ten1 or all three subunits, and subjected sequentially to Ni-NTA and M2 affinity chromatography. The extracts and eluates from the Ni-NTA and M2 resins were analyzed by SDS-PAGE and Coomassie staining. (C) The M2 eluate containing the CST complex from part B was subjected to Glutathione-Sepharose affinity chromatography. The flow through (FT) and eluate fractions as well as the starting material were analyzed by SDS-PAGE and Coomassie staining. (D) Different amounts of the purified CST complex (10 µl, 5 µl and 2.5 µl) and bovine serum albumin (5 µg, 2.5 µg, 1.25 µg, 0.63 µg, and 0.32 µg) were analyzed by SDS-PAGE and Coomassie staining. (E) Serial three fold dilutions of the purified CST complex were subjected to Western analysis using antibodies directed against the SUMO tag, which is present in both the Cdc13 and Stn1 fusion proteins. (F) Purified SUMO-Cdc13 and the CST complex were separately fractionated on 15–30% glycerol gradients. The distributions of the proteins in the fractions were analyzed by Western using antibodies directed against SUMO (the Cdc13 and Stn1 fusions) and GST (the Ten1 fusion protein). The results for free Cdc13 and the CST complex are displayed in the top panel and the bottom three panels, respectively.

To reconstitute the CST complex encoded by the *C. glabrata* genome, we co-expressed all three subunits as fusion proteins in *E. coli* (Figure 1A). The *CDC13*, *STN1* and *TEN1* gene were fused to the FLAG, HIS_6_, and GST tag, respectively to allow sequential affinity purification of the complex. The Cdc13 and Stn1 fusion proteins also contained a SUMO tag, which improved their expression level and solubility. Unless explained otherwise, the fusion proteins will henceforth be referred to as Cdc13, Stn1 and Ten1 to simplify the discussion. Both Ten1 and Cdc13 were recovered from the initial Ni-NTA column, indicating that they can both associate with Stn1 (Figure 1B, lane 4 and 5). The much higher concentrations of Stn1 and Ten1 in comparison to Cdc13 in these fractions are consistent with the expression levels of these proteins (data not shown). As expected, Cdc13 was further enriched after purification on the M2 (anti-FLAG) resin. More importantly, both Stn1 and Ten1 were again recovered, at concentrations that were either equal to Cdc13 (Ten1), or higher than Cdc13 (Stn1) (Figure 1B, lane 7 and 8). Notably, the binding of Stn1 and Ten1 to M2-agarose could not be detected in the absence of Cdc13 (Figure 1B, lane 6). To confirm the formation of the ternary complex, we subjected the M2-derived fractions to Glutathione-Sepharose chromatography and once more recovered all three proteins (Figure 1C). The protein yield from the Glutathione column was low and the composition of the Glutathione fractions was similar to the M2 fractions. Hence, we carried out all subsequent analysis of CST using the M2 fractions.

The apparently higher levels of Stn1 in the CST complex prompted us to carry out a more detailed analysis of the stoichiometry of the complex. First, we quantified the levels of individual proteins by comparing their Coomassie staining intensities to BSA standards (Figure 1D). Assuming that the intensities of Cdc13, Stn1 and Ten1 are proportional to their molecular weights, we obtained a ratio of roughly 1∶2.5∶1 for these subunits in the complex. Second, we estimated the relative amounts of Cdc13 and Stn1 by Western analysis using antibodies directed against the SUMO tag that is present in both fusion proteins. This method again yielded a Cdc13: Stn1 ratio of 1∶2.5 (Figure 1E). In particular, the Western signal of Stn1 in a 3-fold diluted CST sample was slightly less than that of Cdc13 in the undiluted sample (Figure 1E, compare each pair of neighboring samples, e.g., lane 3 & 4). We analyzed multiple CST preparations and found the Stn1: Cdc13 ratio to be always between 2∶1 and 3∶1, and the Ten1: Cdc13 ratio to be nearly 1∶1. We were unable to discriminate firmly between the 2∶1 or 3∶1 stoichiometry for Stn1: Cdc13. It is possible that some heterogeneity exists in the composition of the complex. Alternatively, the staining intensity of Stn1 may not be strictly proportional to its size, thus causing errors in the calculation of molar ratios.

Because Cdc13 is known to dimerize [18], [26], our estimated ratio for the subunits suggests that the complex may have a 2∶4∶2 or 2∶6∶2 stoichiometry, hence a native size of ∼500–600 kDa. Consistent with this notion, glycerol gradient sedimentation analysis of purified CST revealed a peak for each subunit at ∼500 kDa (Figure 1F, the lower 3 panels). Even though the distribution of all three proteins in the gradient are broad, plots of the relative levels of each protein across the gradient are consistent with co-sedimentation around 500 kDa (Figure S1A). A lighter peak for Cdc13 was also observed, implying partial disassembly of the complex during the gradient run. The position of this peak (∼160 kDa) was precisely that predicted for a Cdc13 dimer and was identical to the sedimentation position of purified Cdc13 alone (Figure 1F, the top 2 panels). We also analyzed the Stn1: Cdc13 ratio in the 500 kDa gradient fraction using anti-SUMO antibodies and again obtained a ratio of ∼2.5∶1 (Figure S1B). We conclude that the CST complex has a stoichiometry of 2∶4∶2 or 2∶6∶2.

### Stn1, but not Ten1, can independently form a stable complex with Cdc13

**Figure 2:**
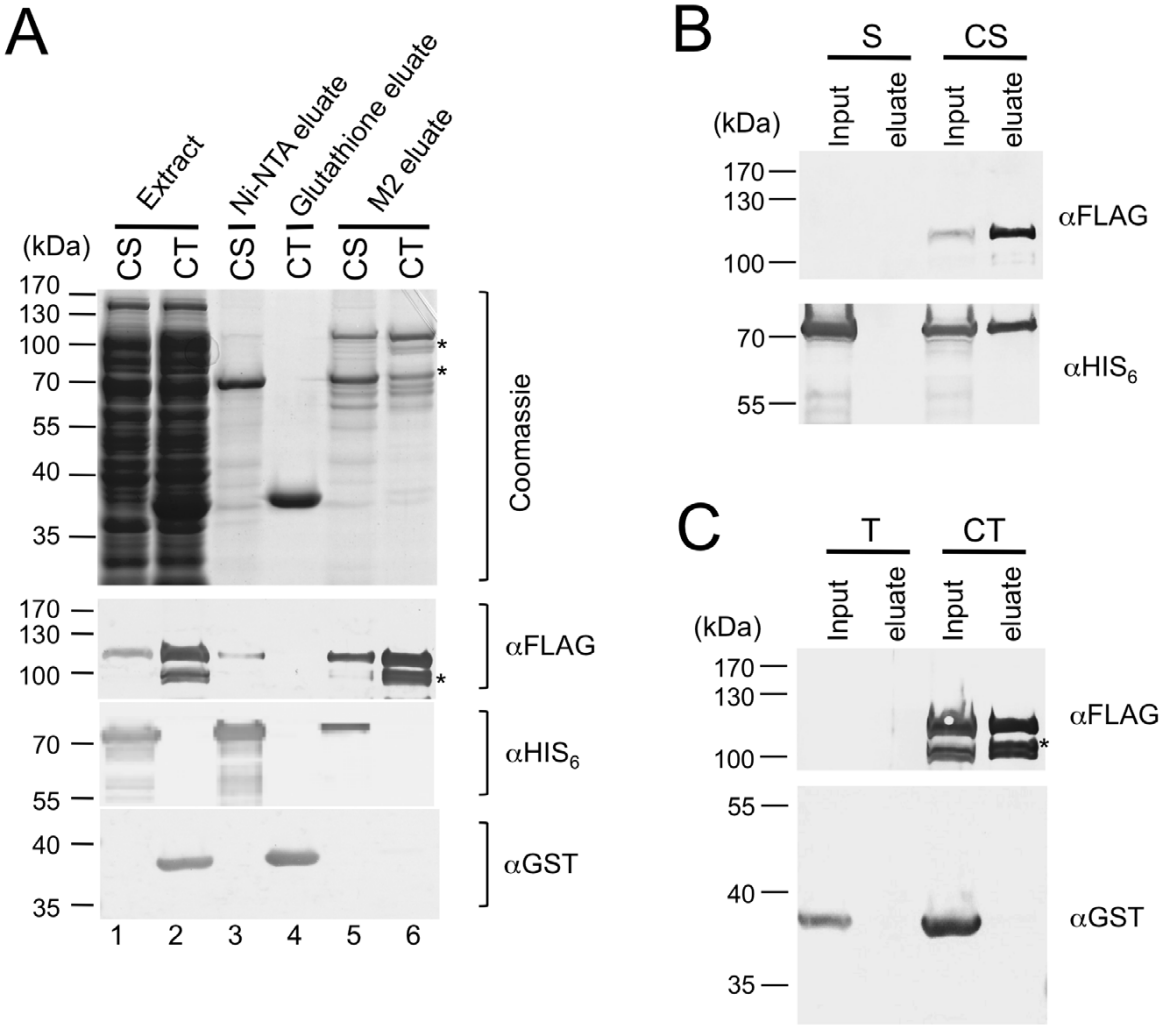
Stn1, but not Ten1, independently binds to Cdc13. (A) *E. coli* extracts prepared from strains co-expressing SUMO-Cdc13-FG and HIS_6_-SUMO-Stn1 (CS) or SUMO-Cdc13-FG and GST-Ten1 (CT) were subjected to Ni-NTA, Glutathione-Sepharose and M2 affinity purification as indicated. The extracts and eluates were analyzed by SDS-PAGE and Coomassie staining (top), as well as Western using antibodies directed against the FLAG tag, the HIS_6_ tag and the GST tag of Cdc13, Stn1 and Ten1 fusion protein, respectively (bottom). Significant amounts of Cdc13 proteolytic fragments (*) can be detected in the CT eluate from the M2 resin. (B) *E. coli* extracts prepared from strains expressing HIS_6_-SUMO-Stn1 alone (S) or HIS_6_-SUMO-Stn1 and SUMO-Cdc13-FG (CS) were subjected to M2 affinity purification. The Cdc13 and Stn1 fusion proteins in the input and eluate samples were detected by Western using antibodies directed against the FLAG and HIS_6_ tag, respectively. (C) *E. coli* extracts prepared from strains expressing GST-Ten1 alone (T) or GST-Ten1 and SUMO-Cdc13-FG (CT) were subjected to M2 affinity purification. The Cdc13 and Ten1 fusion proteins in the input and eluate samples were detected by Western using antibodies directed against the FLAG and GST tag, respectively.

To begin to analyze the assembly mechanisms of the CST complex, we separately co-expressed Stn1 and Cdc13, as well as Ten1 and Cdc13, and then used the same affinity purification strategy to assess complex formation. Interestingly, Cdc13 could be recovered in the Ni-NTA fraction that contained highly purified Stn1, but was not detectable in the Glutathione-Sepharose fraction that contained purified Ten1 (Figure 2A, lane 3 and 4). Conversely, Stn1 but not Ten1 could be recovered in the M2 fractions that were highly enriched for Cdc13 (Figure 2A, lane 5 and 6). These results imply that Stn1 but not Ten1 directly interacts with Cdc13. Because Stn1 was at least 20 fold more abundant than Cdc13 in the extract (data not shown), one would expect the majority of Cdc13 to be associated with Stn1, but not vice versa. Consistent with this expectation, Stn1 was ∼2–3 times more abundant than Cdc13 in the M2 fraction, but was present at far greater excess over Cdc13 in the Ni-NTA fraction (Figure 2A, lane 3 and 5). As further controls, we expressed Stn1 or Ten1 alone in the absence of Cdc13 and subjected the extracts to M2 affinity chromatography (Figure 2B and 2C). Little of either protein was found in the eluate, thus ruling out non-specific binding of Stn1 to the M2 resin. Altogether, these experiments support a model of CST assembly that entails a direct interaction between Cdc13 and Stn1, but not between Cdc13 and Ten1.

### Characterization of the DNA–binding properties of *C. glabrata* Cdc13 and CST complex

**Figure 3:**
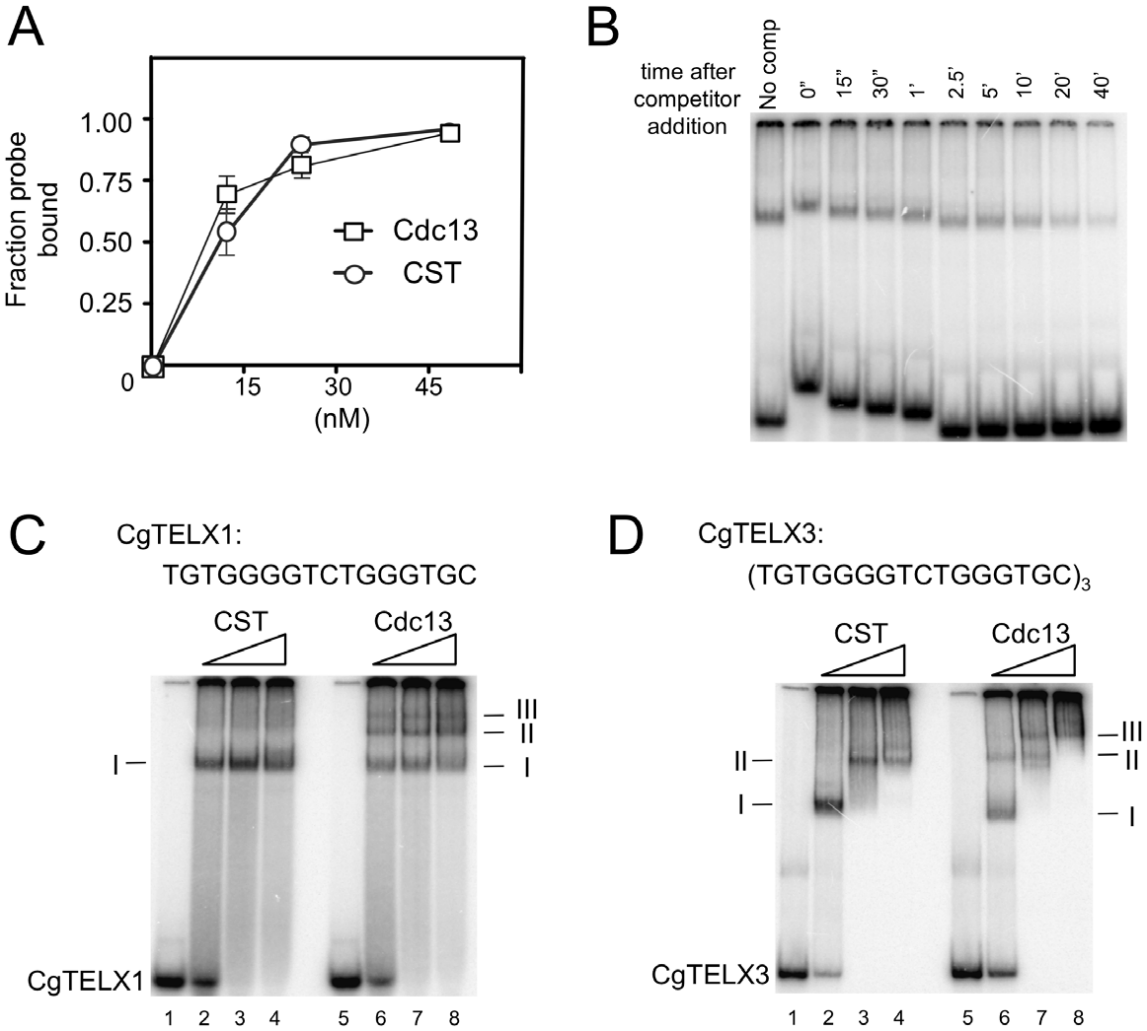
DNA–protein complexes generated by Cdc13 and CST. (A) The apparent K_d_ of Cdc13 and CST for the CgTELX1 probe (TGTGGGGTCTGGGTGC) was estimated in gel shift assays using different concentrations of the protein or protein complex. Data (average ± standard deviation) are from three independent experiments. (B) The stability of the CST-DNA complex was estimated in a competition experiment. CST (10 nM) was allowed to form complexes with labeled CgTELX1 (7.5 nM) for 20 min. One-hundred fold excess unlabeled CgTELX1 was then added, and the mixture applied to a native gel at various time points following the addition of the competitor. The non-uniform mobility of the free probe and the complex was due to the fact that samples were applied at different times. The fractions of labeled complexes remaining at various time points are determined and used to calculate t_½_. The experiments were repeated three times and the estimated t_½_ ranges from 5 to 13 min. (C) Increasing concentrations of CST and Cdc13 (15 nM, 30 nM and 60 nM) were incubated with P^32^-labeled CgTELX1 oligo (7.5 nM) and the resulting complexes analyzed by electrophoresis through a native gel. Complexes of decreasing mobility are designated by I, II, etc. (D) Same as part C except that P^32^-labeled CgTELX3 oligo ([TGTGGGGTCTGGGTGC]_3_) was used as the probe.

We next attempted to characterize the DNA-binding properties of individual subunits or sub-complexes as well as the full CST assembly in order to assess the contributions of these subunits. Among the subunits and sub-complexes, we were able to obtain substantial amounts of Cdc13, Ten1 and the Stn1-Ten1 (ST) sub-complex for gel mobility shift analysis. Neither Ten1 nor ST could form a detectable complex with *C. glabrata* telomere oligonucleotides in a variety of buffer conditions in these assays (data not shown). In contrast, Cdc13 and CST each bound telomere oligoes with a K_d_ of ∼10–20 nM (Figure 3A). The Cdc13-DNA and CST-DNA complexes were relatively stable with a ½ life of ∼10 min (Figure 3B and data not shown). Binding was also highly sequence-specific. In competition assays, more than 200 fold higher concentrations of a random unlabeled oligo (R1) as well as a heterologous telomere oligo (*Le*TELX2, corresponding to 2 copies of the *L. elongisporus* telomere repeat) were needed to achieve the same degree of inhibition as the *C. glabrata* telomere oligo (Figure S2). Interestingly, an oligo based on the *C. lusitaniae* telomere repeat (*Cl*TELX2) exhibited moderate affinity for the *C. glabrata* Cdc13 and CST; just 20-fold higher concentration of this oligo competed as well as the *C. glabrata* telomere repeat (Figure S2).

We then attempted to determine the lengths of DNA occupied by Cdc13 and CST through titration analysis. We reasoned that a fast migrating complex should be converted to a slower complex at higher protein concentrations if the former contains sufficient naked DNA to capture additional proteins. Conversely, if the DNA in a complex is fully occupied, then adding more proteins should not alter its mobility (unless the added proteins can form oligomers with the proteins in the complex). Characterization of the DNA-protein species formed by Cdc13 and CST on a 16-nt (one repeat) probe suggests that both can fully occupy this DNA target (Figure 3C). In the case of CST, only one predominant gel shift band could be detected, and this band was not converted to other forms even at a protein concentration that was twice than necessary to retard all of the free probe. For Cdc13, even though three gel shift bands could be observed, the fastest migrating complex (I) was not converted into other forms even at much higher protein concentrations. This finding suggests that while multiple Cdc13 dimers may be able to land on a 16-nt DNA (Figure 3C, complex II and III), a significant fraction of the probe was fully occupied by just one Cdc13 dimer.

Interestingly, characterization of the complexes formed on a 48-nt probe suggests different occupancy sizes for Cdc13 and CST (Figure 3D). For Cdc13, three complexes could be observed, and the highest mobility species (complex I) was progressively converted to lower mobility species (complex II and III) with increasing Cdc13 concentrations. By contrast, only two species of CST-DNA complex could be detected over the same range of protein concentrations. These observations suggest that the 48-nt probe can be fully occupied by three Cdc13 dimers, but just two CST complexes. Hence the CST complex appears to occupy a longer stretch of G-tail than the Cdc13 dimer. However, the difference is evidently subtle and requires additional studies (e.g., footprinting) for confirmation.

### Binding of CST to telomere G-strand alters DNA conformation

**Figure 4:**
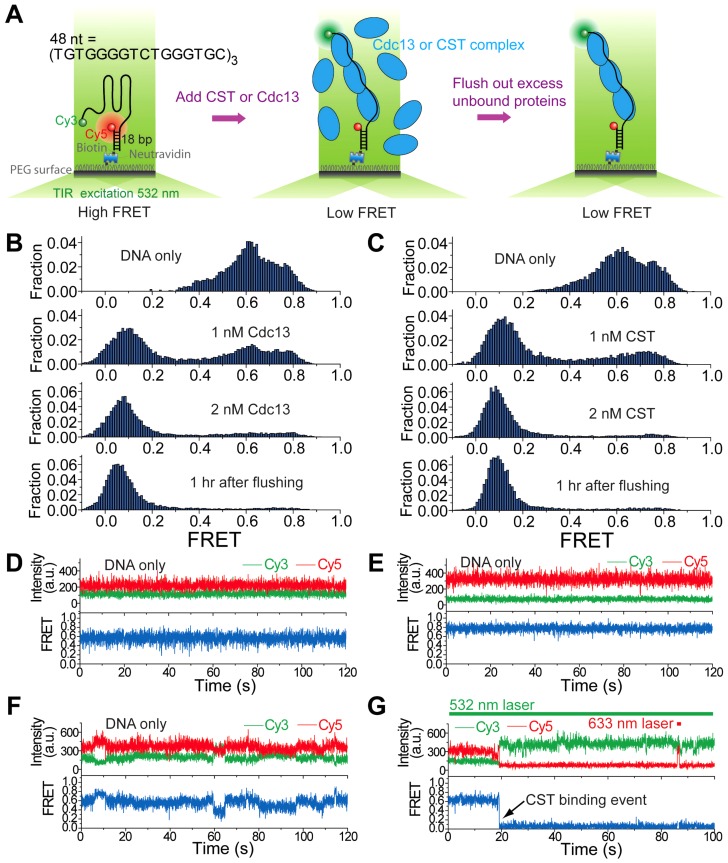
Cdc13 and CST can bind and unfold higher order G-tail structure. (A) A schematic depiction of reaction steps for single molecule FRET experiments. (B) Single molecule FRET efficiency histograms in the presence of the indicated concentrations of Cdc13. (C) Single molecule FRET efficiency histograms in the presence of the indicated concentrations of CST. (D–F) Representative single-molecule FRET-time traces of the 48-nt G-tail construct in 3 mM MgCl_2_ and 100 mM NaCl before adding any protein. A 532 nm laser was used for excitation. (G) A representative single-molecule FRET-time trace of the 48-nt G-tail construct in 3 mM MgCl_2_ and 100 mM NaCl immediately after adding 1 nM CST, showing a CST binding event at ∼19 sec (the black arrow). A 532 nm laser was used for Cy3 excitation throughout the entire course of data acquisition. A 633 nm laser was briefly turned on for direct Cy5 excitation at ∼86 sec in order to confirm that Cy5 is still active rather than photobleached.

G-tails have the propensity to form salt-dependent higher order structures (e.g., G-quadruplex) [27], [28]. Cdc13 and other G-tail binding proteins have been reported to bind or modulate such higher order structures [27], [29], [30], but the activity of the complete CST complex in this regard has not been characterized. We used a single-molecule FRET strategy [31], [32], which has been used to study the dynamics of various proteins on ssDNA [33], to characterize the interaction between *C. glabrata* telomeric DNA and either Cdc13 or CST. The standard DNA construct contains 48 nt of G-tail DNA ([TGTGGGGTCTGGGTGC]_3_) and harbors fluorescent dyes (a donor-acceptor FRET pair) near its 5′ and 3′ ends (Table 1, Figure 4A, and Figure S3A). In the presence of low concentrations of salt (e.g., 10 mM K^+^), the majority of DNA probes appear to be unfolded as indicated by the low FRET efficiencies (∼0.1 to 0.2) (Figure S3B). FRET efficiencies increased steadily in the presence of progressively higher salt concentrations (Figure S3B), consistent with the formation of salt-dependent higher order DNA structures with reduced distance between the fluorescent donor and acceptor [34]. Evidence for higher order G-tail structures can also be gleaned from comparison to the FRET signals generated from a 32-nt G-tail and (dT)_32_ (Figure S4B and S4D). FRET efficiencies for the 32-nt G-tail (∼0.7) was substantially higher than that for (dT)_32_ (∼0.35) despite their identical lengths, suggesting that the G-tail is folded into a compact conformation rather than forming a random coil. Additional evidence suggesting “folded G-tails” came from FRET experiments that utilized Li^+^, which is known to be less efficient than Na^+^ and K^+^ in stabilizing G-quadruplex [35]. Indeed, the single molecule FRET-time traces in 100 mM Li^+^ exhibited greater dynamics than in 100 mM Na^+^, indicating that the folded state is more short-lived in Li^+^ (Figure S4C). Moreover, the peaks of the FRET histogram were consistently lower in Li^+^ than in the same concentrations of K^+^ (e.g., 0.15 in 20 mM Li^+^ and 0.25 in 20 mM K^+^) (Figure S3B and S3D). However, it is important to stress that folded DNA structures other than G-quadruplex are also compatible with our data.

**Table 1:** Oligonucleotides used in the single-molecule FRET experiments.

Oligonucleotide	Sequence
1	5′-/Cy5/GCCTCGCTGCCGTCGCCA -/biotin/- 3′
2	5′- TGGCGACGGCAGCGAGGC(T)_16_/iAmMC6T/(T)_24_ - 3′
3	5′- TGGCGACGGCAGCGAGGC(T)_31_/iAmMC6T/(T) - 3′
4	5′- TGGCGACGGCAGCGAGGCTGTGGGGTCTGGGTGC - 3′
5	5′- TGGCGACGGCAGCGAGGC(TGTGGGGTCTGGGTGC)_2_/Cy3/- 3′
6	5′- TGGCGACGGCAGCGAGGC(TGTGGGGTCTGGGTGC)_3_/Cy3/- 3′

Binding of Cdc13 and CST to the 48-nt G-tail construct was analyzed in solution containing 3 mM MgCl_2_ and 100 mM NaCl. Prior to protein addition, the G-tail was evidently folded and yielded a broad FRET distribution in which at least three high FRET states (∼0.4, ∼0.6 and ∼0.8) can be discerned (Figure 4B and 4C, top panels), similar to the FRET distribution obtained in 500 mM K^+^ (Figure S3B). This implies the existence of several folded conformations for the 48-nt G-tail, which is expected for a G-tail containing six repeats of the ‘GGG’ motif. In single-molecule FRET-time traces, the G-tail molecules show two heterogeneous behaviors: Some G-tails maintain their particular folded conformation for a long duration (Figure 4D and 4E), whereas others show fluctuations between different conformations (Figure 4F). This heterogeneity in the conformational dynamics of single molecules was previously observed for the human telomeric G-tail [34]. The introduction of either Cdc13 and CST resulted in the disappearance of the three high FRET species (∼0.4, ∼0.6 and ∼0.8) and a corresponding increase in low FRET (∼0.1) molecules, indicating that both Cdc13 and CST can bind and disrupt the higher order structures associated with high FRET efficiencies (Figure 4B and 4C, second and third panels). The CST binding event can be observed in single-molecule FRET-time traces as an abrupt decrease in FRET (Figure 4G). Similar shifts from the high FRET to low FRET states were observed when we added CST to two other DNA constructs with G-tail lengths of 16 or 32 nt (Figure S4A and S4B). Notably, we observed an inverse relation between the lengths of G-tail and the FRET efficiencies of protein-bound DNA; the peak FRET efficiencies were ∼0.35, 0.11, and 0.09 for the 16 nt, 32 nt and 48 nt constructs, respectively. This correlation suggests that the CST-bound DNAs adopt an extended conformation.

Similar to the results obtained in the gel mobility shift assays, we found very stable binding of Cdc13 and CST to the G-tail in the FRET experiments. For the 32 nt and 48 nt G-tail constructs, we were unable to detect the reappearance of high FRET DNA molecules even an hour after flushing the system with a protein-free buffer (Figure 4B and 4C, Figure S4B). For the 16 nt G-tail construct, about half of the complexes appear to disassemble 30 min after the application of protein-free buffer (Figure S4A). Thus, maximal stability of the CST-DNA and Cdc13-DNA complex appear to require more than one telomere repeat unit.

### Binding of Cdc13 and CST to non-telomeric DNA

**Figure 5:**
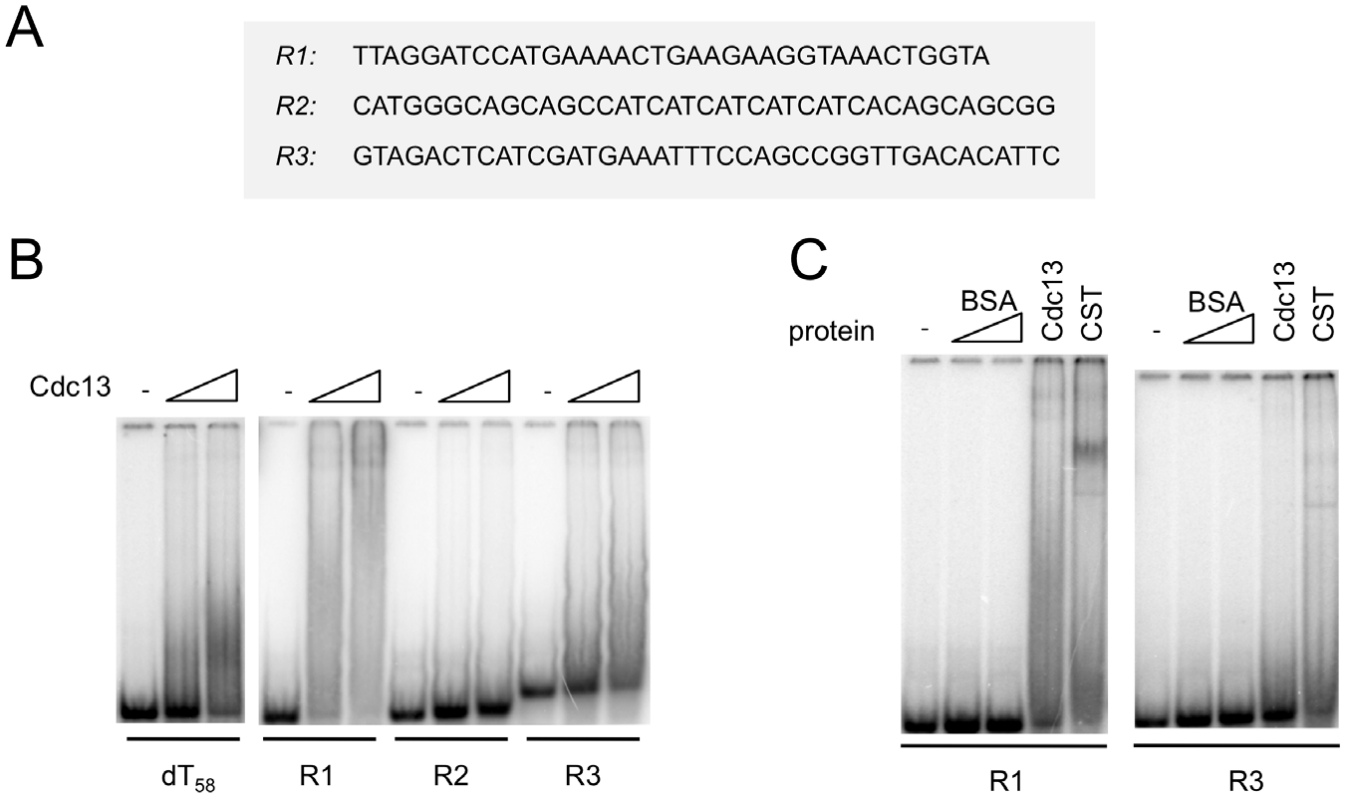
Cdc13 and CST form unstable complexes with non-telomeric DNA. (A) The sequences of the non-telomeric oligonucleotides used in the gel mobility shift assays are displayed. (B) Increasing concentrations of purified Cdc13 (0 nM, 40 nM and 80 nM) were incubated with the indicated oligonucleotide probes (7.5 nM) and the resulting complexes analyzed by gel electrophoresis and PhosphorImager scanning. (C) Labeled R1 and R3 probes (7.5 nM) were incubated with BSA (80 and 240 nM), Cdc13 (40 nM), and CST (40 nM), and the resulting complexes analyzed by gel electrophoresis and PhosphorImager scanning.

During the course of the single molecule FRET analysis, we found that Cdc13 and CST can also alter the FRET of a poly-dT control construct. For example, in one experiment, the addition of 100 nM CST to fluorescent (dT)_58_ (in which the Cy5 and Cy3 fluorophores are ∼16 nt apart) resulted in a shift of FRET from ∼0.6 to ∼0.45 for a significant fraction of the molecules (Figure S5). Importantly, CST binding to this oligonucleotide is evidently unstable; the number of DNA molecules with ∼0.45 FRET efficiency was greatly reduced just 2 minutes after the system was flushed with the binding buffer (Figure S5B and S5C). This observation suggests that Cdc13 and CST may have the ability to bind transiently to non-telomeric DNA, and prompted us to re-investigate this issue using a variety of non-telomeric oligonucleotides in gel mobility shift assays. Indeed, we found that Cdc13 can alter the mobility of the (dT)_58_ as well as several other non-telomeric DNA (Figure 5A and 5B). Notably, the complexes formed on the non-telomeric oligonucleotides did not migrate as discreet species, but rather as broad smears that trail upward from the position of the free probe (Figure 5B and 5C). Such behavior can be explained by gradual dissociation of the complexes during gel electrophoresis, and is hence quite consistent with the FRET findings. Similar binding to non-telomeric DNAs was also observed for the CST complex (Figure 5C and data not shown). These results provide biochemical support for the notion that the CST complex may have the ability to act on non-telomeric DNA *in vivo*.

### *Cg*Stn1 and *Cg*Ten1 have weak intrinsic DNA–binding activities

**Figure 6:**
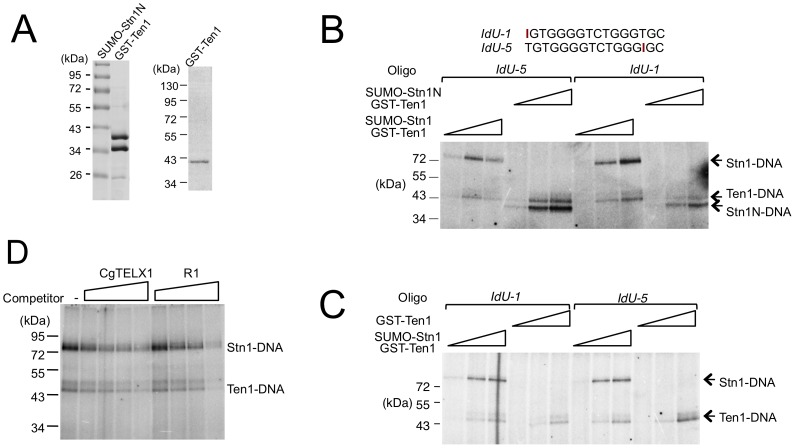
Crosslinking of *C. glabrata* Stn1 and Ten1 to photo-reactive telomere oligonucleotides. (A) Purified protein complex containing SUMO-Stn1N and GST-Ten1 fusion, as well as GST-Ten1 alone, was analyzed by SDS-PAGE and Coomassie staining. (B) Increasing concentrations of the indicated Stn1-Ten1 complexes (6.6 nM, 20 nM, 60 nM) were crosslinked to labeled IdU-1 or IdU-5 oligonucleotides and the covalent products detected by SDS-PAGE and PhosphorImager scanning. (C) Increasing concentrations of the Stn1-Ten1 complex or GST-Ten1 alone (6.6 nM, 20 nM, 60 nM) were crosslinked to labeled IdU-1 or IdU-5 oligonucleotides and the covalent products detected by SDS-PAGE and PhosphorImager scanning. (D) Purified *Cg*Stn1-Ten1 complex (240 nM) was crosslinked to labeled IdU-1 oligonucleotide (16 nM) in the presence of increasing concentrations of the indicated competitor oligonucleotides (125 nM, 500 nM, 2 µM and 8 µM).

The potential difference between the occupancy size of the Cdc13 dimer and CST complex suggests that Stn1 and Ten1 may contact DNA directly in the context of the full CST-DNA assembly. To address this possibility, we first carried out crosslinking studies using purified CST and a telomere oligonucleotide containing an Iodo-dU substitution. Even though putative Stn1-DNA adducts could be detected, the signals for such adducts were quite weak in comparison to the Cdc13-DNA products, making it difficult to draw clear conclusions (data not shown). We therefore repeated the crosslinking assays using just the Stn1-Ten1 subcomplex. Because the N-terminal OB fold of Stn1 is presumed to be the DNA-binding domain, we also tested a Stn1N-Ten1 complex. Analysis of labeled products indicates that both Stn1 and Ten1 can be readily crosslinked to DNA (Figure 6A and 6B). The efficiency of Stn1-DNA or Stn1N-DNA adduct formation was consistently about 3 fold higher than Ten1-DNA. Furthermore, Ten1 alone was capable of direct binding to DNA, and the efficiency of Ten1-DNA crosslinking was not stimulated by its association with Stn1 (Figure 6A and 6C). Interestingly, moving the Iodo-dU substitution in the substrate from the 5′ to the 3′ end did not significantly change the crosslinking efficiency of either protein, suggesting that the proteins may not be positioned in a specific manner in relation to the DNA (Figure 6B and 6C, compare signals generated by IdU-1 and IdU-5). Moreover, in competition assays, a non-telomeric oligonucleotide inhibited the crosslinking efficiency to the same extent as the telomere oligo (Figure 6D). Thus, in the absence of Cdc13, Stn1 and Ten1 appear to bind DNA weakly in a non-sequence specific manner.

### The DNA–binding activity of *C. albicans* Stn1 is required for telomere protection

**Figure 7:**
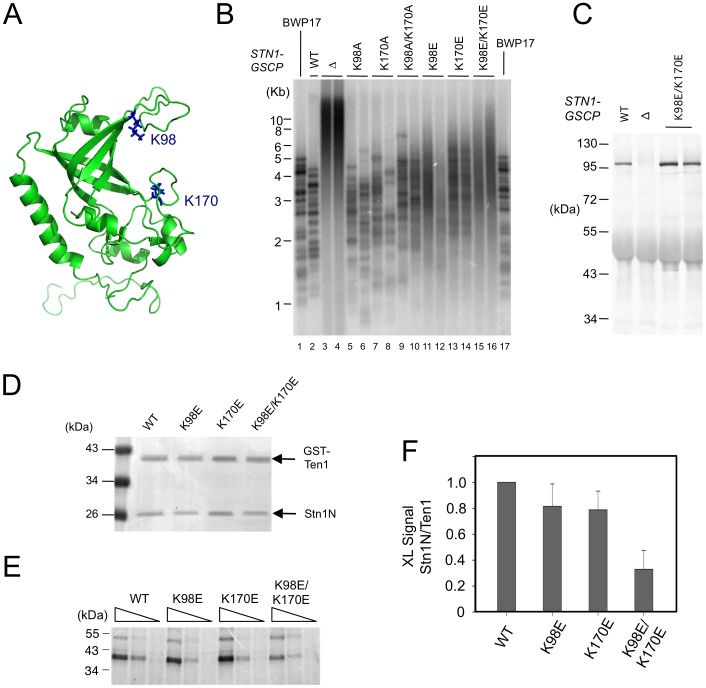
The effects of *C. albicans* Stn1 mutations on telomere lengths and Stn1–DNA crosslinking. (A) A homology model of *C. albicans* Stn1N is displayed with two putative DNA-binding residues highlighted in blue. (B) The distributions of telomere restriction fragments in the indicated strains were analyzed by Southern using a probe that corresponds to two copies of the *C. albicans* telomere repeat unit. (C) The levels of *Ca*Stn1-GSCP proteins in extracts derived from the indicated strains were analyzed by Western using antibodies against protein A. (D) Purified complexes containing *C. albicans* GST-Ten1 and the indicated Stn1N (wild type or mutant) were analyzed by SDS-PAGE and Coomassie staining. (E) Different concentrations of the complexes shown in D (300 nM, 100 nM, 33 nM) were crosslinked to a *C. albicans* telomere oligonucleotide containing a single Iodo-dU substitution. The covalent products were detected by SDS-PAGE and PhosphorImager scanning. (F) The levels of crosslinked products for each Stn1N protein were normalized against that for the corresponding GST-Ten1 protein and plotted. Data (average ± standard deviation) are derived from three independent experiments.

The weak and non-sequence specific DNA binding activities of *Cg*Stn1 and Ten1 are consistent with a domain-swapping study, in which the OB fold of *Sc*Stn1 was shown to function in place of the DNA-binding domain of *Sc*Rpa2 [5]. To investigate the relevance of this activity in telomere protection, we mutated potential DNA-binding residues in *C. albicans* Stn1 and tested the effects of mutations on telomere length regulation. Inspection of a homology model of *Ca*Stn1 revealed two basic residues (K98 in L_12_ and K170 in L_45_) that are well positioned to contact DNA directly (Figure 7A). We mutated these residues separately or together to either Ala or Glu, and tested the ability of the resulting alleles to complement the *stn1* null mutant. As described in a previous report, the telomeres of the null mutant are substantially longer and more heterogeneous than the parental strain (Figure 7B, lane 3–4) [6]. Re-integrating a tagged but otherwise wild type *STN1* gene (*STN1-GSCP*) suppressed the telomere defects (Figure 7B, lane 2) [6]. Interestingly, the Ala substitution mutants (K98A, K170A and K98A/K170A) exhibited little or no defects in telomere regulation; discreet telomere fragments can still be detected in the mutants and only the K98A/K170A combination mutant manifested a slight increase in telomere lengths (Figure 7B, lane 5–10). In contrast, all of the Glu mutants exhibited significant increases in their telomere lengths and heterogeneity, with the combination mutant (K98E/K170E) manifesting the most severe defect (Figure 7B, lane 11–16). The dysfunction of the Stn1 mutants cannot be attributed to diminished protein levels (Figure 7C and data not shown). In addition, we found that K98E/K170E mutant exhibited normal binding to *C. albicans* Cdc13 in a co-expression/co-immunoprecipitation analysis (Figure S6). Thus, we surmise that the negative charges introduced by the Glu mutations probably rendered Stn1 defective in telomere protection by disrupting the direct contacts between Stn1 and DNA.

To confirm that the Stn1 mutants are indeed defective in DNA-binding, we purified and analyzed *C. albicans* ST complexes containing either wild type or mutated *Ca*Stn1N. Because the His_6_-SUMO-*Ca*Stn1N and GST-*Ca*Ten1 fusion proteins expressed in *E. coli* exhibited very similar mobility in SDS-PAGE, we removed the His_6_-SUMO tag before subjecting the complex to the photo-crosslinking assay (Figure 7D). In accordance with the telomere length defects described earlier, the K98E and K170E single mutants each exhibited a mild reduction in crosslinking efficiency, whereas the double mutant manifested a more severe, 3 fold reduction (Figure 7E and 7F). The correlation between the *in vivo* and *in vitro* phenotypes suggests that the DNA-binding activity of *Ca*Stn1 is required for full telomere protection.

## Discussion

The chief significance of the current work resides in the discovery of an unanticipated subunit arrangements for the CST complex and the functional characterization of the DNA-binding properties of individual subunits and complexes. The mechanistic and evolutionary implications of these findings are discussed below.

### CST subunit arrangement is different from the RPA complex

Not withstanding the strong similarities between Stn1-Ten1 and Rpa2-Rpa3, previous studies have revealed two major differences between Cdc13 and Rpa1. First, the available high-resolution structures of Cdc13 OB fold domains do not resemble those of Rpa1 [18], [26], [36]. Second, homo-dimerization appears to be a common feature of Cdc13, but not of Rpa1. The current study discloses additional differences between the CST and RPA complex with respect to subunit stoichiometry and inter-subunit interactions. Specifically, RPA has a well-characterized 1∶1∶1 stoichiometry that is established by a trio of interacting α helices, one from each of the subunits [37]. CST, on the other hand, are probably assembled through separate Cdc13-Stn1 and Stn1-Ten1 interactions (this work and [6]). The higher copy number of Stn1 additionally suggests the existence of multiple Stn1-binding sites on a single Cdc13 molecule. Clearly more structural studies are necessary to delineate the molecular basis of CST assembly, but existing data already points a different organizing principle for CST from RPA. The higher Stn1 copy number also raises the intriguing possibility of functional specialization, i.e., different Stn1 in the same complex may bind to different targets (e.g., Pol α and DNA) to mediate distinct functions. Of the three CST components, Stn1 is clearly the best conserved at the level of amino acid sequence [5], suggesting greater evolutionary constraint that could be due to a large number of binding targets.

### Stn1 has a weak but functionally significant DNA–binding activity

Previous investigations of DNA-binding by Stn1 and Ten1 have yielded disparate results. Whereas the *S. cerevisiae* Stn1 and Ten1 were shown individually to bind G-rich telomeric DNA with moderate to low affinity, the *C. tropicalis* Stn1-Ten1 failed to form detectable complexes with the corresponding telomere repeats [5], [6]. The current report provides a potential explanation for this discrepancy. Even though the *C. glabrata* Stn1-Ten1 complex or Ten1 protein did not show evidence of interaction with telomeric DNA in native gels, they can clearly make direct contacts to DNA as judged by the photo-crosslinking analysis. Moreover, our discovery of comparable DNA-crosslinking by *C. albicans* Stn1-Ten1 strongly supports the notion that DNA-binding is a conserved (and hence functionally important) property of these proteins. This notion was presaged by an earlier analysis of the *Drosophila* orthologue *Verrocchio*; a quadruple substitution on the putative DNA-binding surface of this protein caused telomere dysfunction [12]. In the current study, we found a good correlation between the DNA-binding defects of Stn1 mutants and telomere dysfunction, thus providing further support for the physiologic significance of this activity. However, we cannot exclude the possibility that the DNA-binding mutants are deficient in some other aspects of Stn1 function. The notion of direct-DNA contacts by Stn1 and Ten1 extends further the parallel between the RPA and CST complex in terms of the mechanisms of DNA-binding. Even though the two smaller subunits of each complex have very low intrinsic affinity for single-stranded DNAs, they probably do touch DNAs directly in the context of the full assembly ([38] and this work).

### The CST complex can unfold higher order G-tail structures

Higher order G-tail structures can inhibit the action of telomerase and other nucleic acid enzymes, and the propensity of *C. glabrata* G-tails for G-quadruplex formation is well documented [27], [28]. Previous investigations suggest that one function of G-tail binding proteins such as Pot1 and Cdc13 is to unfold such structures to facilitate important telomere transactions [27], [29], [30]. However, all such earlier studies utilized individual G-tail binding proteins rather than the more physiologically relevant complexes. Here we show that the *C. glabrata* CST complex can indeed unfold higher order G-tail structures as judged by single-molecule FRET analysis. The near complete loss of DNA FRET signals upon protein binding, together with the inverse relationship between FRET efficiencies and G-tail lengths, suggests that the G-tail may adopt a relatively extended conformation after the loading of CST complex. In this respect, the yeast G-tail coated by CST may be structurally distinct from the mammalian G-tail coated by the corresponding binding complex known as POT1-TPP1, which appears to form a compact and potentially more ordered structure [39]. Yeast telomere G-tails are known to undergo S-phase specific lengthening due to the resection of the complementary C-strand [40], [41]. The long G-tails generated during the S-phase presumably have a strong propensity to form folded structures that may pose a challenge for nucleic acid enzymes. Indeed, helicases such as Pif1 and Sgs1 have been proposed to help the cell unwind problematic G-quadruplex structures [42], [43]. The significance of the G-tail unfolding activity of the CST complex in relation to quadruplex-unwinding helicases, and whether there is coordination between these activities are important issues worthy of further investigation.

### *C. glabrata* Cdc13 and CST can bind non-telomeric single-stranded DNA with moderate affinity

One of our surprising observations is the ability of Cdc13 and CST to form unstable complexes with non-telomeric DNA. Even though in competition assays, the protein and the complex exhibited at least 200-fold preference for the cognate telomere repeat, they are able to bind with moderate affinity to some non-telomeric DNAs of similar sizes in the absence of the preferred target sequence. This observation recalls analysis of other OB fold proteins such as the ciliate TEBP, which was found to be capable of using different molecular contacts to bind non-cognate target DNAs [44]. The recognition mechanisms of *C. glabrata* CST for telomeric and non-telomeric DNAs are likely to be distinct given the dramatic differences in target sequences and in the stability of the complexes. It is tempting to speculate that the non-telomeric DNA-binding activity of the *C. glabrata* CST complex may allow it to mediate non-telomeric functions. Hints that yeast CST may mediate non-telomeric functions came from the observation that over-expression of Stn1 engenders HU sensitivity and checkpoint defects [45]. Evidence for non-telomeric activities of the mammalian complex is even stronger. For example, there is incomplete co-localization of mammalian CST and telomere DNA in the nucleus, and knockdown of CTC1 results in DNA-damage foci both at and away from telomeres [10], [11]. In addition, the human disease(s) caused by CTC1 mutations (known as Coats plus or CRMCC) has a phenotypic spectrum that is distinct from the prototypical telomere diseases such as dyskeratosis congenita [46], [47]. Finally, the human CST complex was recently implicated in promoting replication re-start after replication stress [17]. Along this line, it is worth noting that the mammalian CST complex has been shown to bind long ssDNA of non-telomere sequences [10], [14]. Our discovery of a non-telomeric DNA binding activity of the *Cg*CST complex suggests a greater degree of mechanistic conservation of the yeast and mammalian complex than previously realized.

## Materials and Methods

### Construction and growth of *Candida* strains

The *C. albicans* laboratory strain *BWP17* (*ura3Δ::λimm434/ura3Δ::λimm434 his1::hisG/his1::hisG arg4::hisG/arg4::hisG*) and the *stn1* null derivative have been described previously [6], [48]. To analyze the functions of *Ca*Stn1 DNA-binding mutants *in vivo*, the relevant mutations were introduced into the integrating plasmid pGEM-URA3-STN1-GSCP (containing an epitope tagged and functional *STN1* allele and the *URA3* selectable marker) by QuikChange mutagenesis [6]. The resulting plasmids were linearized by Hpa I digestion and then used to transform the *C. albicans stn1* null mutant. Correct integration of the linearized DNA in the transformants was confirmed by Southern analysis.

### Telomere analyses

The telomere length analysis was performed as previously described [49].

### Expression and purification of CST subunits and complexes

The following plasmids were constructed in order to express *C. glabrata* CST subunits and complexes: pSMT3-*Cg*CDC13-FG, encoding a HIS_6_-SUMO-*Cg*Cdc13-FLAG fusion protein; pACYC1-DUET-*Cg*CDC13-FG, encoding a SUMO-*Cg*Cdc13-FLAG fusion; pSMT3-*Cg*STN1, encoding a HIS_6_-SUMO-*Cg*Stn1 fusion; and pGEX-6P1-*Cg*TEN1, encoding a GST-Ten1 fusion. Individual plasmids or combinations thereof were transformed into BL21(DE3), and the strains were cultured in LB and induced for protein expression with IPTG as described earlier [26]. Following induction, extracts were prepared and the expressed protein or protein complexes purified by appropriate combinations of affinity chromatography as follows: HIS_6_-SUMO-*Cg*Cdc13-FG, Ni-NTA-agarose and M2-agarose columns; the CST complex, Ni-NTA-agarose and M2-agarose columns; the Stn1-Ten1 (ST) sub-complex, Ni-NTA-agarose and glutathione-Sepharose columns. The Ni-NTA and Glutathione-Sepharose chromatography procedures were performed as previously described. For purification on the M2 resin, the starting extracts or fractions were adjusted to the composition of the FLAG binding buffer (50 mM Tris-HCl (pH 7.5), 150 mM NaCl, 2.5 mM MgCl_2_, 1 mM DTT, 0.1% NP-40, and 10% Glycerol) and incubated with 1/10 to 1/20 vol. of M2-Agarose resin at 4°C on a rotator for 3 h. The resin was then washed 5 times with the FLAG binding buffer, and eluted with FLAG binding buffer containing 0.2 mg/ml 3XFLAG peptide.

For the analysis of *C. albicans* Stn1N-Ten1 complex, PCR fragments encoding *Ca*Stn1N (amino acid 1–239) and *Ca*Ten1 were inserted into the pSMT3 and pGEX-4T2 vectors to allow for the expression of HIS_6_-SUMO-*Ca*Stn1N and GST-*Ca*Ten1 fusion proteins, respectively. The complex was synthesized in *E. coli* and isolated by tandem affinity chromatography as described above for the *C. glabrata* Stn1-Ten1 complex. The roles of the K98 and K170 residues of Stn1 in DNA-binding were tested by mutating the corresponding codons in pSMT3-*Ca*STN1N to either Ala or Glu. Mutant complexes were made and analyzed using the same method as that described for the wild type complex.

### Gel electrophoretic mobility shift analysis

Binding reactions contained 10 mM Tris-HCl (pH 8.0), 2 mM MgCl_2_, 1 mM spermidine, 1 mM DTT, 10% Glycerol, 7.5 nM oligonucleotide probe (∼80,000 c.p.m), and the indicated concentrations of purified proteins in 20 µl total volume. Following incubation at 22°C for 20 m, the reaction mixtures were electrophoresed through a 5% nondenaturing polyacrylamide gel (acrylamide/bis-acrylamide 44: 1) to resolve the free probe from the DNA-protein complex. Binding activity was analyzed using a Typhoon PhosphorImager and the ImageQuant software (GE Healthcare).

### UV crosslinking assay

Crosslinking reactions were conducted in 12 µl mixtures that included 50 mM Hepes-NaOH, pH 7.5, 10% glycerol, 30 mM K-acetate, 2 mM Mg-acetate, 1 mM DTT, 2 nM 5′ P^32^-labeled oligonucleotide containing a single Iodo-dU substitution (∼50,000 c.p.m.), and indicated concentrations of proteins. The binding between the proteins and the oligonucleotide was allowed to proceed at 22°C for 10 min, followed by 20 min of UV irradiation (Model UVM-57, UVP Inc.) on ice. The conjugates were separated in SDS-PAGE (10–11%) and detected by PhosphorImager analysis.

### Single-molecule FRET experiments

The oligonucleotides used in the FRET experiments are listed in Table 1. The amine-modified thymine (/iAmMC6T/) shown in the sequence enables the oligonucleotides to be labeled with the monofunctional NHS ester form of Cy3 dyes (GE Healthcare)./Cy5/represents the Cy5 dye that was inserted directly to the DNA backbone using phosphoramidite chemistry. The partial duplex DNA substrates (18 bp dsDNA) with single-stranded tails carrying fluorescence dyes were generated by annealing ∼5 µM of biotinylated strand and ∼7 µM of the longer strand in 10 mM Tris-HCl (pH 8.0) and 50 mM NaCl, followed by slow cooling from 90°C to room temperature for ∼2 hours.

All single-molecule measurements were performed at 22±1°C. 50–100 pM of the annealed DNA substrates were immobilized on a quartz slide surface which is coated with polyethyleneglycol (mPEG-SC, Laysan Bio) in order to eliminate nonspecific surface adsorption of proteins [50], [51]. The immobilization was mediated by biotin-Neutravidin binding between biotinylated DNA, Neutravidin (Pierce), and biotinylated polymer (Bio-PEG-SC, Laysan Bio). Next, an imaging buffer containing 20 mM Tris-HCl (pH 7.5), 3 mM MgCl_2_, 100 mM NaCl, 1 mM DTT, 0.1 mg/ml BSA, 2% (v/v) glycerol, 0.5% (w/v) D-glucose, 165 U/ml glucose oxidase, 2170 U/ml catalase, 3 mM Trolox and with desired CST or Cdc13 concentrations was directly added and incubated with the surface-tethered DNA substrates for 5 min before data acquisition. A total internal reflection fluorescence (TIRF) microscope previously described [32] was used for data acquisition. Finally, the same imaging buffer (but with no protein) was used to flush out the excess unbound protein from solution for another round of data acquisition. Single-molecule FRET-time traces was recorded with a time resolution of 30 ms and the FRET histograms were generated by averaging for 300 ms. To study the monovalent ion induced G-tail structures for the 48-nt three telomere repeats, desired concentration of KCl was used to replace 3 mM MgCl_2_ and 100 mM NaCl in the imaging buffer.

### Homology modeling

The homology model of *Ca*Stn1N was calculated using the I-Tasser server (http://zhanglab.ccmb.med.umich.edu/I-TASSER/) with 3KF8.pdb (the structure file for *Ct*Stn1N) specified as the template. The resulting model was visualized and analyzed in Pymol (http://pymol.sourceforge.net/index.html).

## Supporting Information

Figure S1 Analysis of CST complex in glycerol gradient fractions. (A) The relative levels of Cdc13, Stn1 and Ten1 in the glycerol gradient fractions shown in Figure 1F were determined and plotted. The gradient analysis was repeated three times and the ∼500 kDa peaks for all three subunits are detected in each analysis. (B) Serial two fold dilutions of the 500 kDa glycerol gradient fraction were subjected to Western analysis using antibodies directed against the SUMO tag to estimate the relative levels of Cdc13 and Stn1. (C) Serial two fold dilutions of the 160 kDa glycerol gradient fraction were subjected to Western analysis using antibodies directed against the SUMO tag to estimate the relative levels of Cdc13 and Stn1. The higher level of Cdc13 in this fraction is consistent with the presence of free Cdc13 dimers.
(PDF)

Figure S2 Cdc13 and CST exhibit strong preference for the cognate telomere repeat sequence. (A) The sequences of the oligonucleotides used in the gel mobility shift assays are listed. (B) CST (10 nM) was incubated with P^32^-labeled *Cg*TELX1 probe (7.5 nM) in the presence of increasing concentrations of four different competitor oligonucleotides and the resulting complex detected by gel electrophoresis and PhosphorImager analysis. The molar ratios of the competitor to the probe are 4, 16, and 64 for the *Cg*TELX1 competitor, and 64, 256, 1024 for the other three oligoes. In this and two other series of assays, approximately 20 fold higher concentration of *Cl*TELX2 and more than 200 fold higher concentration of *Le*TELX2 and R1 are needed to achieve the same degree of competition as *Cg*TELX1. (C) Cdc13 (6 nM) was incubated with P^32^-labeled *Cg*TELX1 probe (7.5 nM) in the presence of increasing concentrations of four different competitor oligonucleotides and the resulting complex detected by gel electrophoresis and PhosphorImager analysis. The molar ratios of the competitor to the probe are 4, 16, and 64 for the *Cg*TELX1 competitor, and 64, 256, 1024 for the other oligoes. In this and two other series of assays, approximately 20 fold higher concentration of *Cl*TELX2 and more than 200 fold higher concentration of *Le*TELX2 and R1 are needed to achieve the same degree of competition as *Cg*TELX1.
(PDF)

Figure S3 The fluorescent telomeric G-tail exhibits salt-dependent changes in single molecule FRET histograms. (A) Schematic diagram of the DNA construct used. A partial duplex DNA containing a 3′ 48-nt telomeric G-tail ([TGTGGGGTCTGGGTGC]_3_) was used as in Figure 4. (B) Single molecule FRET efficiency histograms for the 48-nt G-tail at the indicated concentrations of K^+^. (C) Representative single-molecule FRET-time traces for the 48-nt G-tail in 3 mM MgCl_2_ and 100 mM KCl. (D) Single molecule FRET efficiency histograms for the 48-nt G-tail at the indicated concentrations of Li^+^. (E) Representative single-molecule FRET-time traces for the 48-nt G-tail in 3 mM MgCl_2_ and 100 mM LiCl.
(PDF)

Figure S4 Cdc13 and CST alter the FRET of 16-nt and 32-nt G-tails. (A) The structure of the 16-nt G-tail construct is illustrated. Cy3 and Cy5 are attached near the two ends of the 16-nt G-tail. FRET efficiencies of individual 16-nt G-tail molecules were analyzed before and after the addition of 16 nM CST, and at 3 min and 30 min after flushing the system with buffer containing 3 mM MgCl_2_ and 100 mM NaCl. The fraction of molecules displaying particular FRET signals are plotted against the FRET values. (B) The structure of the 32-nt G-tail construct is illustrated. Cy3 and Cy5 are attached near the two ends of the 32-nt G-tail. FRET efficiencies of individual 32-nt G-tail molecules were analyzed before and after the addition of 16 nM CST, and at 1 hr after flushing the system with buffer containing 3 mM MgCl_2_ and 100 mM NaCl. The fraction of molecules displaying particular FRET signals are plotted against the FRET values. (C) Representative single-molecule FRET-time traces for the 32-nt G-tail in 3 mM MgCl_2_ and in either 100 mM NaCl (left) or 100 mM LiCl (right). (D) The structure of a (dT)_32_ construct. Cy3 and Cy5 are attached near the two ends of the 32-nt poly-dT ssDNA. FRET efficiencies of individual (dT)_32_ molecules in buffer containing 3 mM MgCl_2_ and 100 mM NaCl are plotted.
(PDF)

Figure S5 Cdc13 and the CST complex alter the FRET efficiencies of a poly-dT DNA construct. (A) The structure of the 58-nt poly-dT DNA construct ((dT)_16+42_) as well as the experimental protocol is illustrated. Cy3 and Cy5 are separated by 16 nt. (B) FRET efficiencies of individual (dT)_16+42_ molecules were analyzed before and after the addition of 100 nM Cdc13, and after flushing the system with buffer containing 3 mM MgCl_2_ and 100 mM NaCl. The fraction of molecules displaying particular FRET signals are plotted against the FRET values. (C) The FRET signals from individual (dT)_16+42_ molecules were analyzed before and after the addition of 100 nM CST, and after flushing the system with buffer containing 3 mM MgCl_2_ and 100 mM NaCl. The fraction of molecules displaying particular FRET signals are plotted against the FRET values.
(PDF)

Figure S6 The *C. albicans* Stn1-Cdc13 interaction is not affected by the K98E/K170E mutation in Stn1. HIS_6_-SUMO-*Ca*Stn1 fusion proteins (wild type and the K98E/K170E mutant) were expressed alone or in combination with GST-*Ca*Cdc13-FG in *E. coli*. Extracts were prepared from the strains and subjected to M2 affinity purification. The extracts and eluates were analyzed by SDS-PAGE and Coomassie staining (top), as well as Western using antibodies directed against the FLAG tag and the HIS_6_ tag of the Cdc13 and Stn1 fusion protein, respectively (bottom). The putative Cdc13 and Stn1 fusion proteins in the Coomassie stained gel were identified based on co-migration with Western bands and marked by arrows.
(PDF)
