# Supplementary figures and images for: The Telomere Capping Complex CST Has an Unusual Stoichiometry, Makes Multipartite Interaction with G-Tails, and Unfolds Higher-Order G-Tail Structures
Source: PLoS Genet. 2013 Jan 3;9(1):e1003145. doi: 10.1371/journal.pgen.1003145 (PMC3536697; doi:10.1371/journal.pgen.1003145)

Figure S1

A

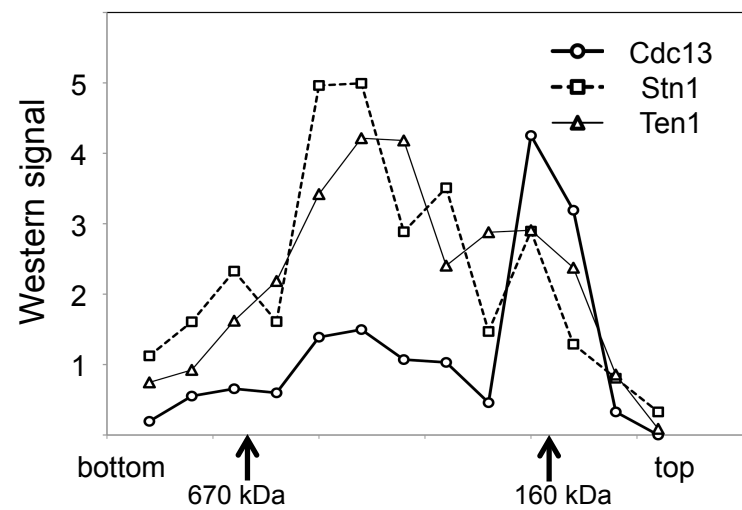

B

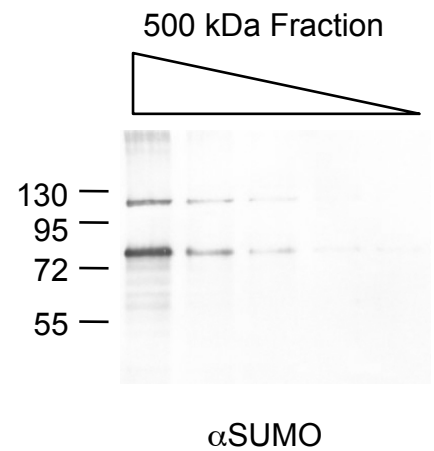

C

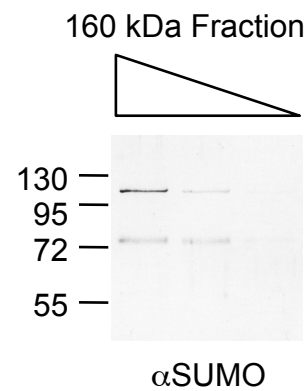

Supplement: Figure S1 — Analysis of CST complex in glycerol gradient fractions. (A) The relative levels of Cdc13, Stn1 and Ten1 in the glycerol gradient fractions shown in Figure 1F were determined and plotted. The gradient analysis was repeated three times and the ∼500 kDa peaks for all three subunits are detected in each analysis. (B) Serial two fold dilutions of the 500 kDa glycerol gradient fraction were subjected to Western analysis using antibodies directed against the SUMO tag to estimate the relative levels of Cdc13 and Stn1. (C) Serial two fold dilutions of the 160 kDa glycerol gradient fraction were subjected to Western analysis using antibodies directed against the SUMO tag to estimate the relative levels of Cdc13 and Stn1. The higher level of Cdc13 in this fraction is consistent with the presence of free Cdc13 dimers. (PDF) [file pgen.1003145.s001.pdf]

Figure S3

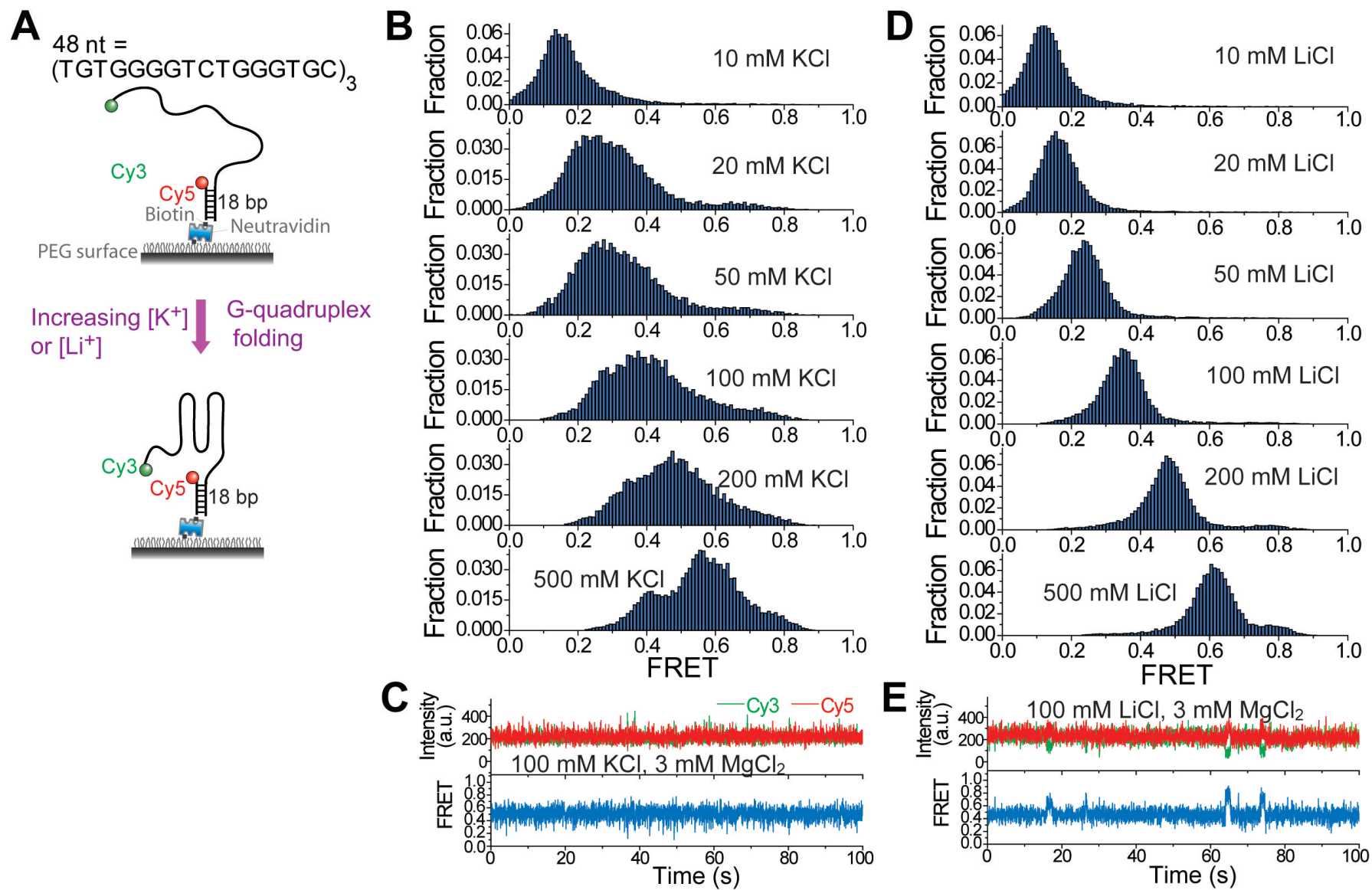

Supplement: Figure S3 — The fluorescent telomeric G-tail exhibits salt-dependent changes in single molecule FRET histograms. (A) Schematic diagram of the DNA construct used. A partial duplex DNA containing a 3′ 48-nt telomeric G-tail ([TGTGGGGTCTGGGTGC]3) was used as in Figure 4. (B) Single molecule FRET efficiency histograms for the 48-nt G-tail at the indicated concentrations of K+. (C) Representative single-molecule FRET-time traces for the 48-nt G-tail in 3 mM MgCl2 and 100 mM KCl. (D) Single molecule FRET efficiency histograms for the 48-nt G-tail at the indicated concentrations of Li+. (E) Representative single-molecule FRET-time traces for the 48-nt G-tail in 3 mM MgCl2 and 100 mM LiCl. (PDF) [file pgen.1003145.s003.pdf]

Figure S4

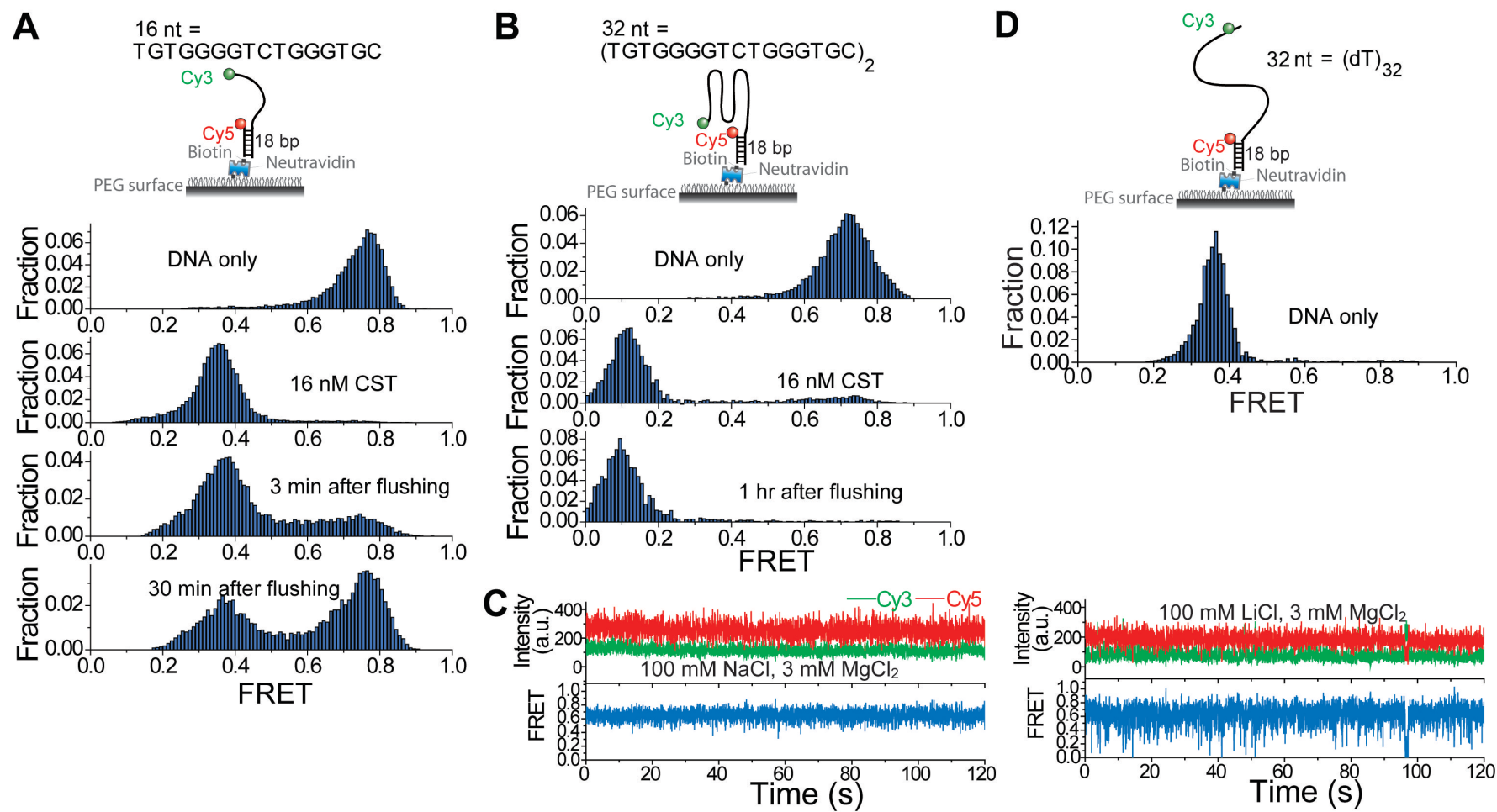

Supplement: Figure S4 — Cdc13 and CST alter the FRET of 16-nt and 32-nt G-tails. (A) The structure of the 16-nt G-tail construct is illustrated. Cy3 and Cy5 are attached near the two ends of the 16-nt G-tail. FRET efficiencies of individual 16-nt G-tail molecules were analyzed before and after the addition of 16 nM CST, and at 3 min and 30 min after flushing the system with buffer containing 3 mM MgCl2 and 100 mM NaCl. The fraction of molecules displaying particular FRET signals are plotted against the FRET values. (B) The structure of the 32-nt G-tail construct is illustrated. Cy3 and Cy5 are attached near the two ends of the 32-nt G-tail. FRET efficiencies of individual 32-nt G-tail molecules were analyzed before and after the addition of 16 nM CST, and at 1 hr after flushing the system with buffer containing 3 mM MgCl2 and 100 mM NaCl. The fraction of molecules displaying particular FRET signals are plotted against the FRET values. (C) Representative single-molecule FRET-time traces for the 32-nt G-tail in 3 mM MgCl2 and in either 100 mM NaCl (left) or 100 mM LiCl (right). (D) The structure of a (dT)32 construct. Cy3 and Cy5 are attached near the two ends of the 32-nt poly-dT ssDNA. FRET efficiencies of individual (dT)32 molecules in buffer containing 3 mM MgCl2 and 100 mM NaCl are plotted. (PDF) [file pgen.1003145.s004.pdf]

Figure S5

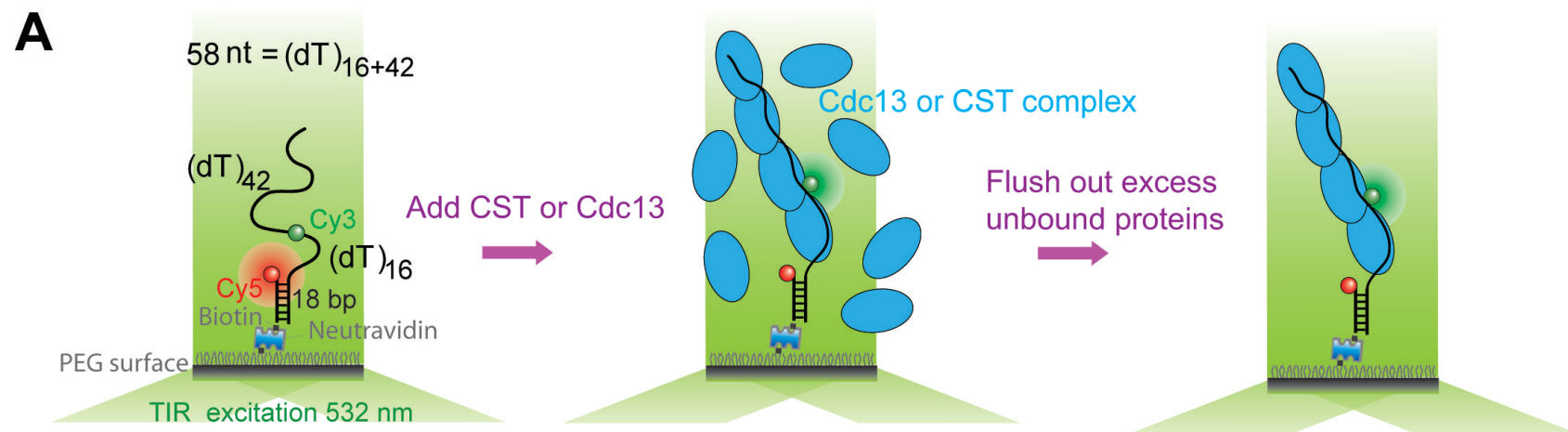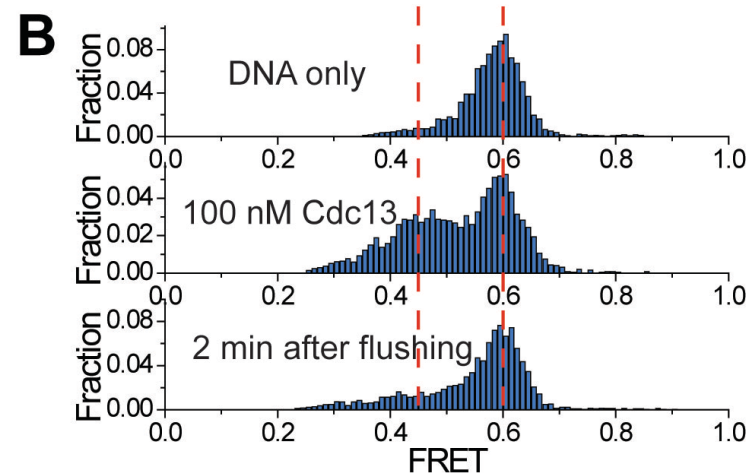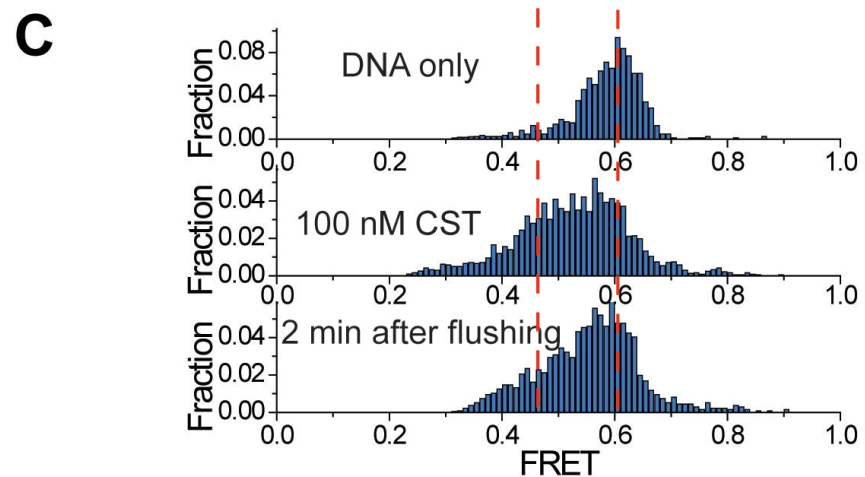

Supplement: Figure S5 — Cdc13 and the CST complex alter the FRET efficiencies of a poly-dT DNA construct. (A) The structure of the 58-nt poly-dT DNA construct ((dT)16+42) as well as the experimental protocol is illustrated. Cy3 and Cy5 are separated by 16 nt. (B) FRET efficiencies of individual (dT)16+42 molecules were analyzed before and after the addition of 100 nM Cdc13, and after flushing the system with buffer containing 3 mM MgCl2 and 100 mM NaCl. The fraction of molecules displaying particular FRET signals are plotted against the FRET values. (C) The FRET signals from individual (dT)16+42 molecules were analyzed before and after the addition of 100 nM CST, and after flushing the system with buffer containing 3 mM MgCl2 and 100 mM NaCl. The fraction of molecules displaying particular FRET signals are plotted against the FRET values. (PDF) [file pgen.1003145.s005.pdf]

# Figure S6

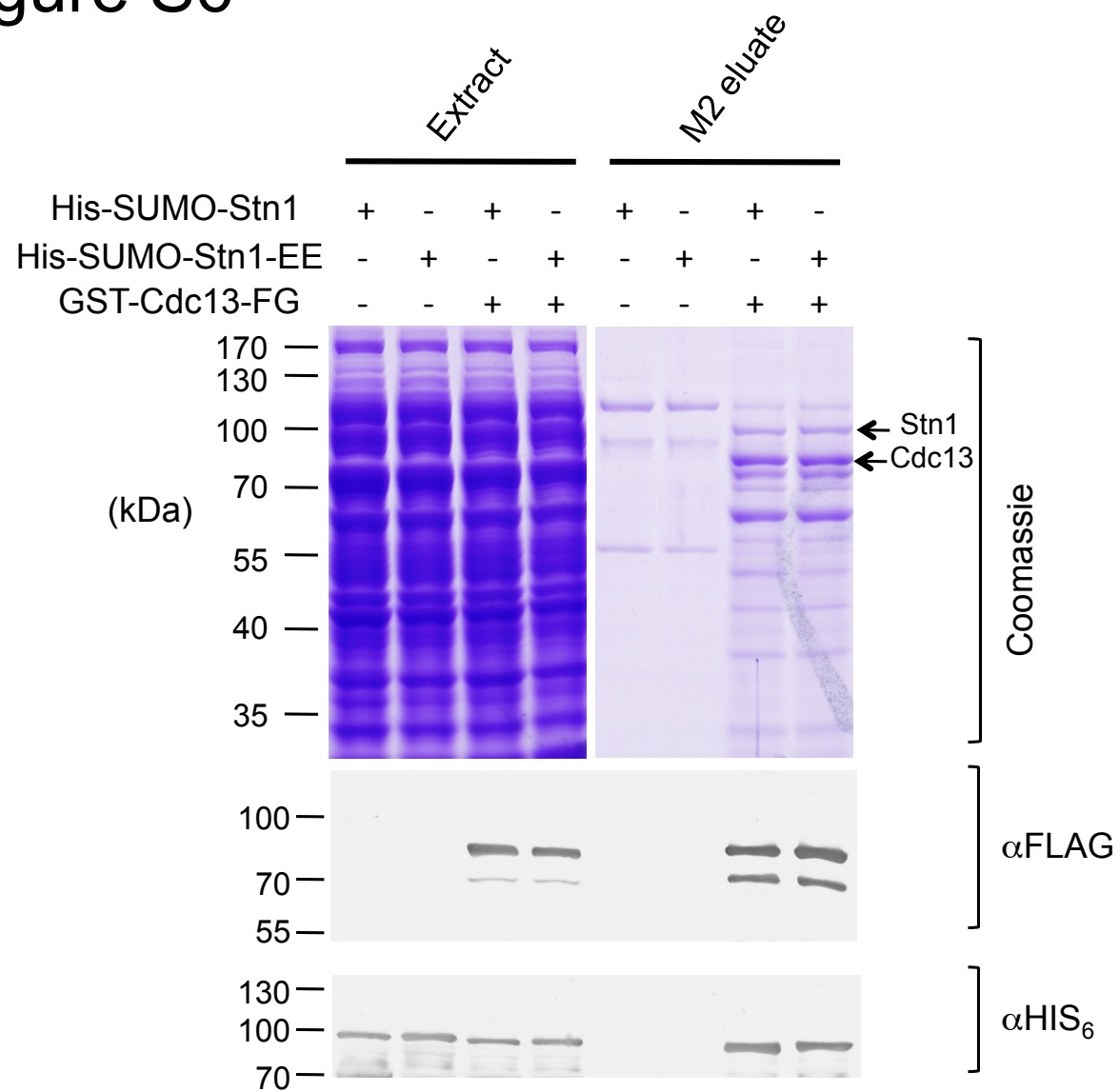

Supplement: Figure S6 — The C. albicans Stn1-Cdc13 interaction is not affected by the K98E/K170E mutation in Stn1. HIS6-SUMO-CaStn1 fusion proteins (wild type and the K98E/K170E mutant) were expressed alone or in combination with GST-CaCdc13-FG in E. coli. Extracts were prepared from the strains and subjected to M2 affinity purification. The extracts and eluates were analyzed by SDS-PAGE and Coomassie staining (top), as well as Western using antibodies directed against the FLAG tag and the HIS6 tag of the Cdc13 and Stn1 fusion protein, respectively (bottom). The putative Cdc13 and Stn1 fusion proteins in the Coomassie stained gel were identified based on co-migration with Western bands and marked by arrows. (PDF) [file pgen.1003145.s006.pdf]
