# Supplementary material for: The Telomere Capping Complex CST Has an Unusual Stoichiometry, Makes Multipartite Interaction with G-Tails, and Unfolds Higher-Order G-Tail Structures
Source: PLoS Genet. 2013 Jan 3;9(1):e1003145. doi: 10.1371/journal.pgen.1003145 (PMC3536697; doi:10.1371/journal.pgen.1003145)

Figure S2

A

*Cg*TELX1	TGTGGGGTCTGGGTG
*Cl*TELX2	(TCTTTAGGGAGGTACTGATGT)$_2$
*Le*TELX2	(CGGTGTAAGGATGCACTTGAAACT)$_2$
R1	TTAGGATCCATGAAAACTGAAGAAGGTAAACTGGTA

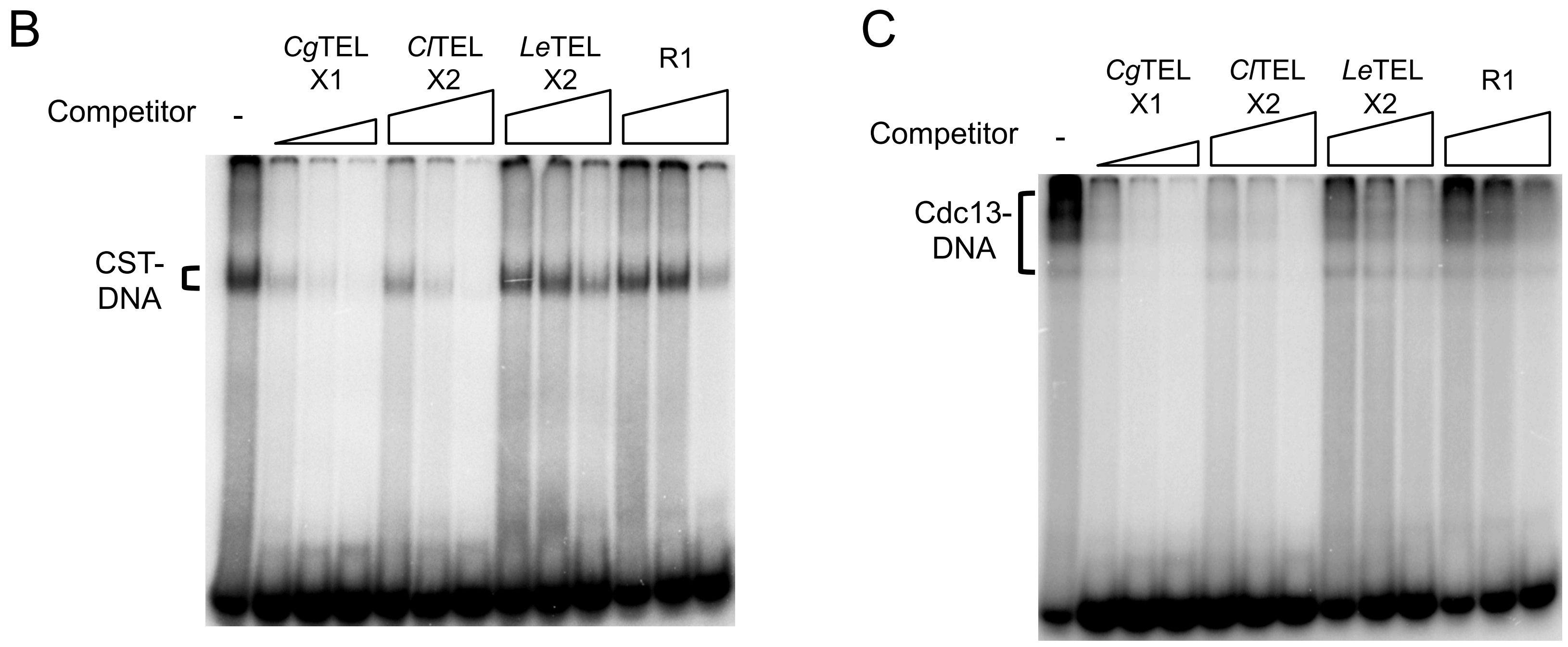

Supplement: Figure S2 — Cdc13 and CST exhibit strong preference for the cognate telomere repeat sequence. (A) The sequences of the oligonucleotides used in the gel mobility shift assays are listed. (B) CST (10 nM) was incubated with P32-labeled CgTELX1 probe (7.5 nM) in the presence of increasing concentrations of four different competitor oligonucleotides and the resulting complex detected by gel electrophoresis and PhosphorImager analysis. The molar ratios of the competitor to the probe are 4, 16, and 64 for the CgTELX1 competitor, and 64, 256, 1024 for the other three oligoes. In this and two other series of assays, approximately 20 fold higher concentration of ClTELX2 and more than 200 fold higher concentration of LeTELX2 and R1 are needed to achieve the same degree of competition as CgTELX1. (C) Cdc13 (6 nM) was incubated with P32-labeled CgTELX1 probe (7.5 nM) in the presence of increasing concentrations of four different competitor oligonucleotides and the resulting complex detected by gel electrophoresis and PhosphorImager analysis. The molar ratios of the competitor to the probe are 4, 16, and 64 for the CgTELX1 competitor, and 64, 256, 1024 for the other oligoes. In this and two other series of assays, approximately 20 fold higher concentration of ClTELX2 and more than 200 fold higher concentration of LeTELX2 and R1 are needed to achieve the same degree of competition as CgTELX1. (PDF) [file pgen.1003145.s002.pdf]
